# Supplementary material for: Fast Super-Resolution Imaging with Ultra-High Labeling Density Achieved by Joint Tagging Super-Resolution Optical Fluctuation Imaging
Source: Sci Rep. 2015 Feb 10;5:8359. doi: 10.1038/srep08359 (PMC4322366; doi:10.1038/srep08359)
Supplement: Supplementary Information [file srep08359-s1.pdf]

Supplementary Information

Fast Super-Resolution Imaging with Ultra-High Labeling Density Achieved by Joint Tagging Super-Resolution Optical Fluctuation Imaging

Zhiping Zeng[1], Xuanze Chen[1], Hening Wang[1], Ning Huang[2], Chunyan Shan[2], Hao Zhang[1], Junlin Teng[2] & Peng Xi[1]*

[1]Department of Biomedical Engineering, College of Engineering, Peking University, Beijing, China;

[2]College of Life Sciences, Peking University, Beijing, China

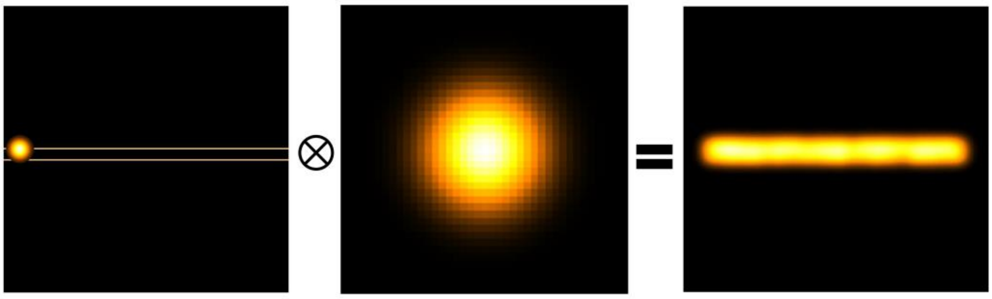

FIGURE S1 Simulated two closely spaced parallel lines (within diffraction limit) convolved with a Gaussian point spread function, generating a blurred image in which two parallel lines cannot be resolved anymore. This simulation illustrates the image formation originating from a diffraction-limited optical system. Each emitting point on these two parallel lines becomes a light spot on the image plane, thereby hindering us from discerning the fine structures.

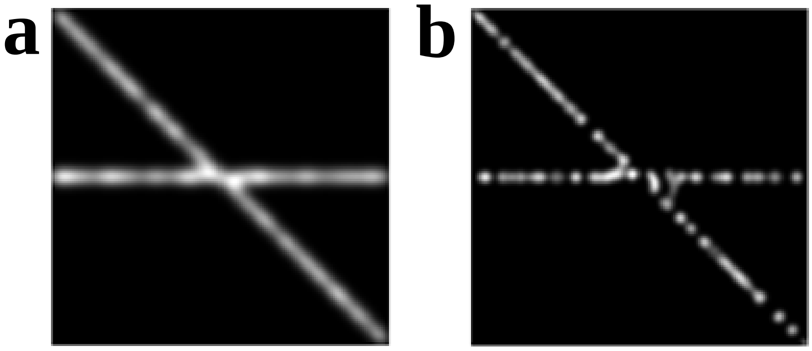

FIGURE S2 Simulation of two cross lines with high labeling density (24 μm^{-1}) blinking emitters. And only 100 time slots are simulated. (a) The 2nd order SOFI presents less

resolution improvement, but the two cross lines are continuous. (b) The 4th order SOFI achieves better resolution enhancement, meanwhile, discontinuities have also been undesirably generated. Image size: 8 µm × 8 µm. Pixel sizes are 40 nm for (a) and 20 nm for (b), respectively.

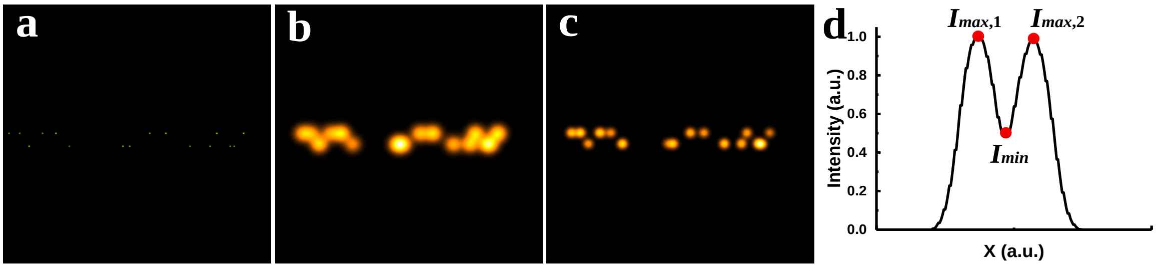

FIGURE S3 Schematic illustration of the visibility defined in Eq. (1). (a) The target distribution. (b) The average image of the targets. (c) 3rd order SOFI image of the targets. (d) Line profile obtained by projecting the SOFI image along horizontal direction. $I_{max,1}$ and $I_{max,2}$ are two normalized peak values along the profile. I_{min} is normalized minimum value along the profile.

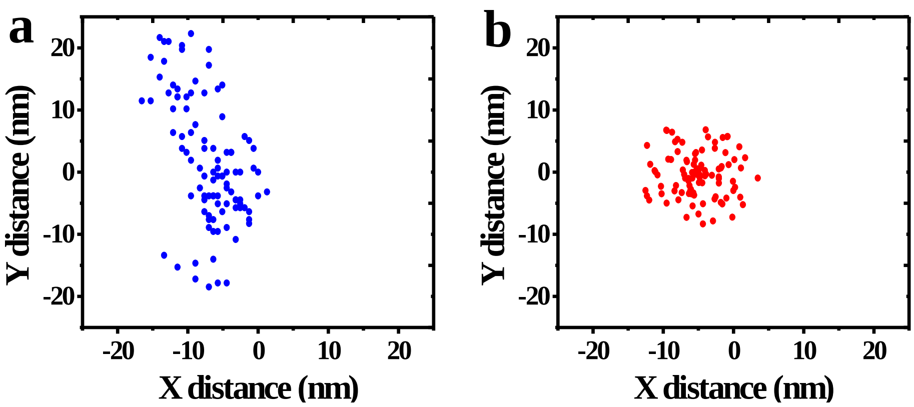

FIGURE S4 Before (a) and after (b) system drift correction of 100 frames. The subpixel drift correction algorithm is able to correct the system drift based on the raw data[1]. No fiducial bead is needed for drift correction. During the experiment, the system drift was caused by various factors, e.g., instability of the stage, vibrations from peripheral equipment and machinery, other unobservable disturbances, etc. Before correction, the drift of the system is indicated by blue dots in (a). As can be seen, the maximum drift along Y direction is over 40 nm. After correction, the drift can be confined within a range of less than 15 nm, indicated by red dots in (b).

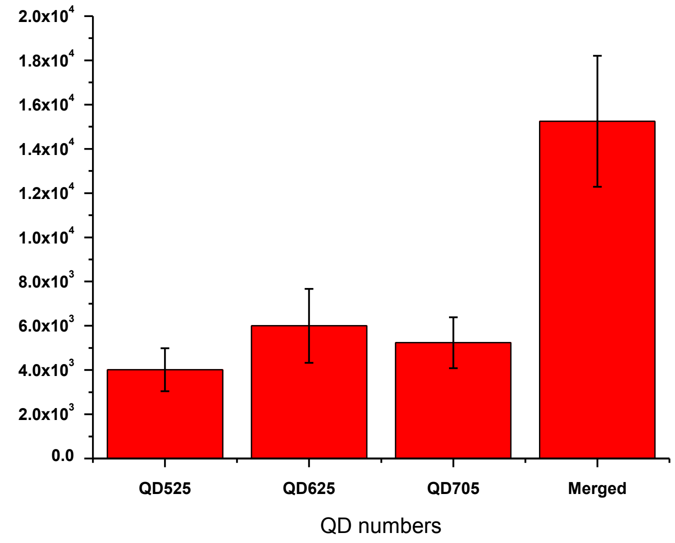

FIGURE S5 Estimated numbers of quantum dots in Figure 3e, 3f, 3g and 3h, respectively.

Since the estimated numbers of QDs in three channels range from roughly 4000 to 6000, and the estimated total lengths of the microtubules range from 136 μm to 149 μm. Thus, taking into account of the volumetric distribution of the QDs labelled to the microtubules, we estimated the density in each color channel is around 7.3 μm^{-1} to 10.1 μm^{-1}.

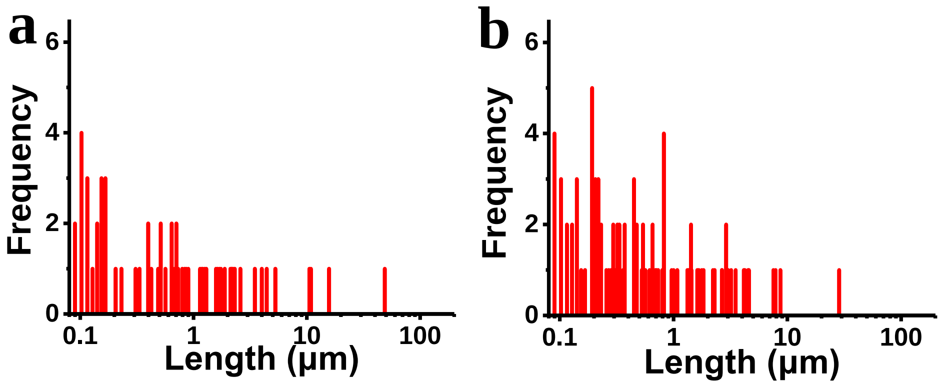

FIGURE S6 (a) Histogram from the skeletonized image shown in Figure 3f. (b) Histogram from the skeletonized image shown in Figure 3g.

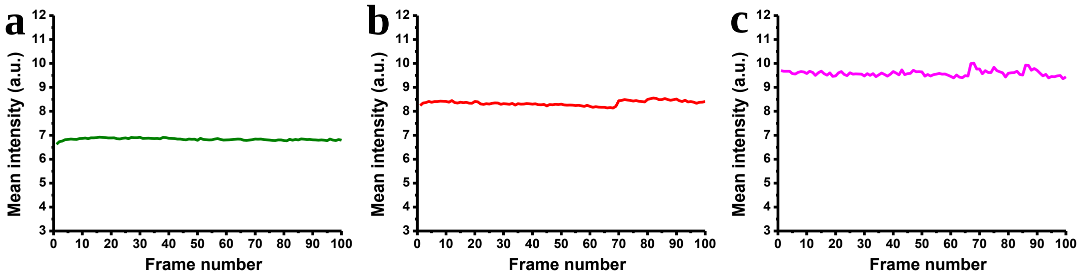

FIGURE S7 Mean fluorescence intensity variations versus frame numbers for (a) QD525, (b) QD625 and (c) QD705. The photobleaching is negligible after capturing 100 frames for all three types of QDs. Due to the photostability of the QDs, photobleaching is not noticeable.

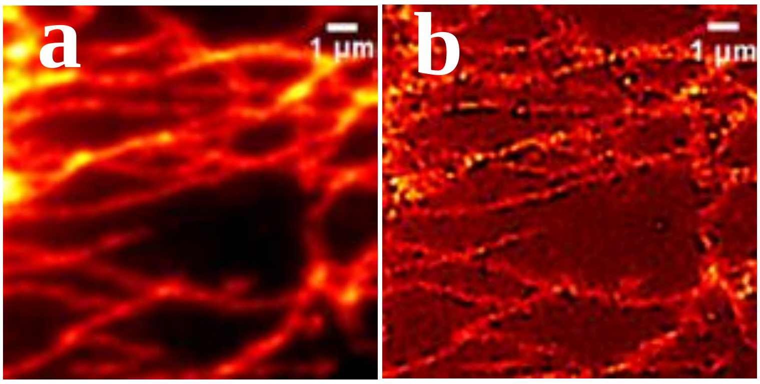

FIGURE S8 Comparison of single color labeled microtubules in possible maximum density. (a) Averaged image. (b) Conventional 4th order SOFI image.

Fig. S8 shows the microtubule networks under the labelling density of 24 μm^{-1}. Fig. S8a is averaged image, Fig. S8b is the conventional 4th order SOFI result. As can be seen, under such high labelling density, without the implementation of JT-SOFI, 4th order SOFI fails to present an acceptable high-resolution image. The generated image suffers from severe artifacts and discontinuities rather than high-quality super-resolution image that JT-SOFI has demonstrated.

JT-SOFI can decrease the requirement for large frame numbers in SOFI processing, thereby improving spatial-temporal resolution simultaneously.

Increasing the frame number can significantly improve the connectivity, as the more frame number, the chances of ON states are also increased linearly. To demonstrate the effect of high-order SOFI processing with different labeling densities and frames, we have simulated the parallel lines with distance of 200 nm, and labeling density of 8 μm^{-1}(upper) and 24 μm^{-1}(lower), respectively, as shown in Fig. S9. When the density is relatively low (8 μm^{-1}), 100 frames for reconstruction is able to present reliable super-resolution image. Whereas, when the density is high (24 μm^{-1}), images generated using less frame numbers (e.g., 100 and 200 frames) contain significant discontinuities. Only images reconstructed using sufficient frame numbers (e.g., 500 and 1000 frames) preserve the continuities of structure. As a consequence, the frame number required for SOFI reconstruction is significantly dependent on the labeling density. Low density labeling is capable of dramatically reducing the frame number, notably expediting the imaging speed for SOFI imaging.

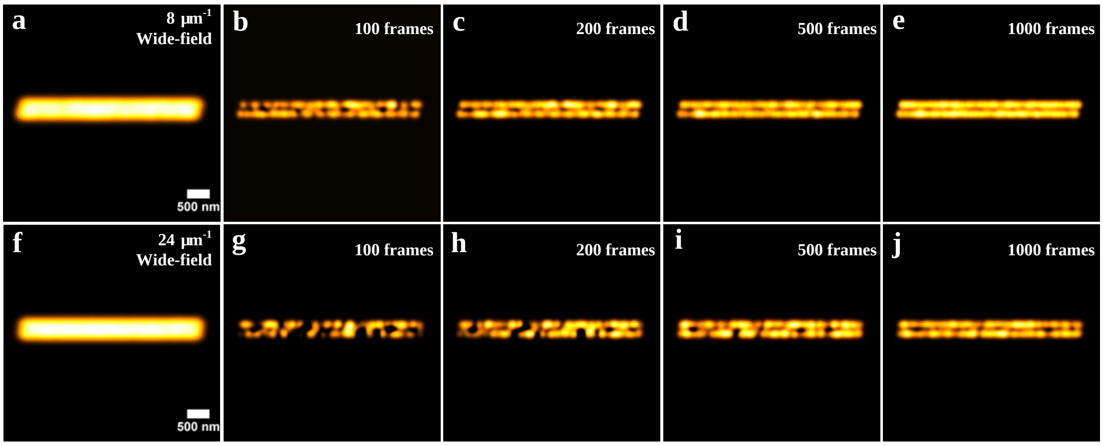

FIGURE S9 (a) Average image of two closely spaced parallel lines distributed with blinking emitters with the labeling density of 8 μm^{-1}. (b-e) 3rd order SOFI images using 100, 200, 500 and 1000 frames for reconstruction, respectively, for labeling density of 8 μm^{-1}. (f) Average image with the labeling density of 24 μm^{-1}. (g-j) 3rd order SOFI images using 100, 200, 500 and 1000 frames for reconstruction, respectively, for labeling of 24 μm^{-1}. *Scale bars: 500 nm.*

In an extreme case, we have also simulated the SOFI reconstruction with only 10 frames, in different labeling density situations, as shown in Fig. S10. The upper and lower panels are for labeling density of 12 μm^{-1} and 3 colors with each 4 μm^{-1}, respectively. As the labeling density is low, the 10-frame averaged wide-field result for 12 μm^{-1} (Fig. S10a) is better than that of 4

μm^{-1} (Fig. S10d). However, the 3-channel JT-SOFI result (Fig.10f) demonstrates even better connectivity than the 12 μm^{-1}, 100 frames result (Fig. S10c).

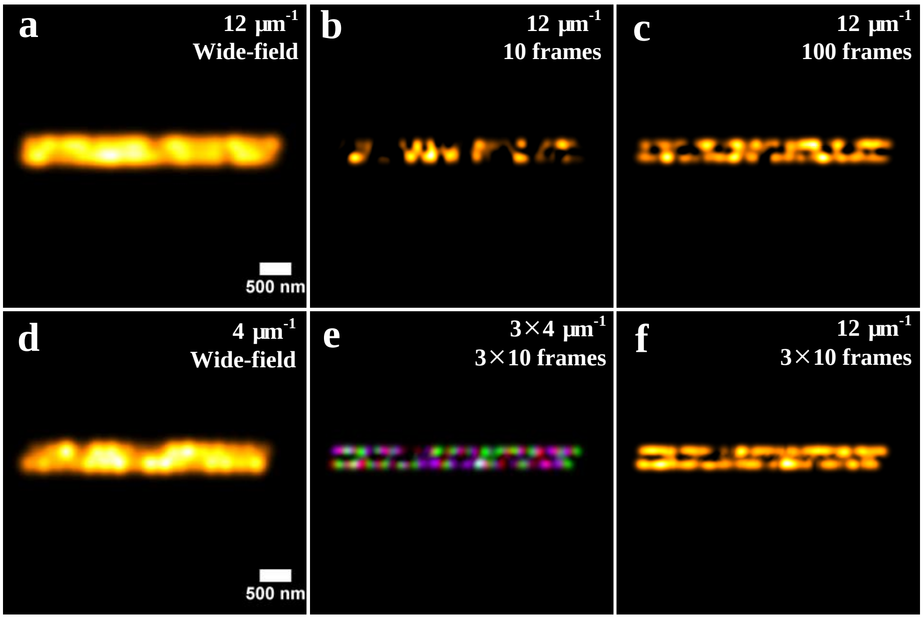

FIGURE S10 (a) Average wide-field image of two closely spaced parallel lines distributed with blinking emitters with the labeling density of 12 μm^{-1}. (b) 3rd order SOFI image using 10 frame for reconstruction with the labeling density of 12 μm^{-1}. (c) 3rd order SOFI image using 100 frame for reconstruction with the labeling density of 12 μm^{-1}. (d) Average wide-field image with the labeling density of 4 μm^{-1}. (e) 3-color joint tagging with the labeling density of 4 μm^{-1} for single channel and 10 frames were used for SOFI reconstruction for each channel. (f) 3rd order SOFI image after summing up 3 channels with the overall labeling density of 12 μm^{-1}. ***Scale bars: 500 nm.***

In Fig. S11, we simulated images with different pixel sizes, at labeling density of 8 μm^{-1}. As smaller pixel size can detect more fluctuation, while larger pixel size averages the fluctuation over space, it can enable SOFI imaging using significantly reduced frame numbers (200 frames) with better image quality (Fig. S11c) over the counterparts with larger pixel size, and 1000 frames (Fig. S11j).

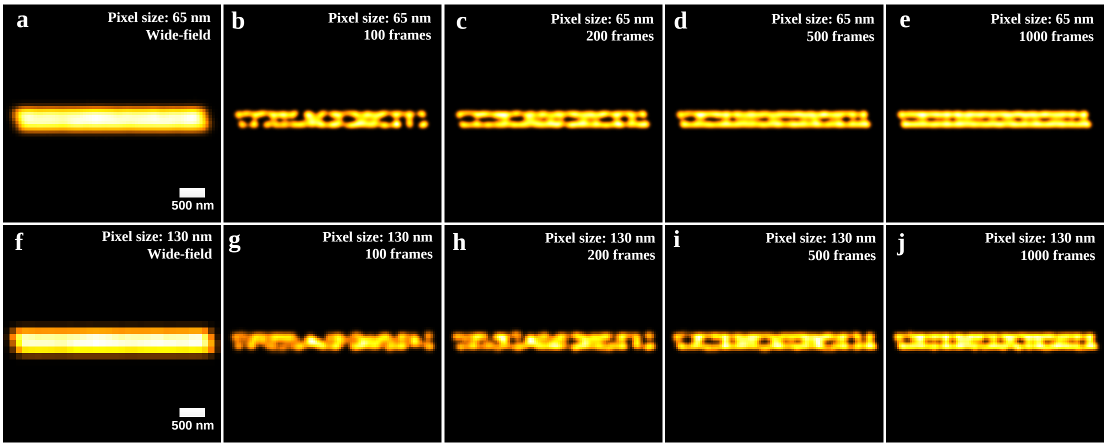

FIGURE S11 (a) Average image of two closely spaced parallel lines distributed with blinking emitters with the pixel size of 65 nm. (b-e) 3rd order SOFI images using 100, 200, 500 and 1000 frames for reconstruction, respectively, for pixel size of 65 nm. (f) Average image with the pixel size of 130 nm. (g-j) 3rd order SOFI images using 100, 200, 500 and 1000 frames for reconstruction, respectively, for pixel size of 130 nm. *Scale bars: 500 nm.*

Simulation of real-time imaging of living cells.

In this simulation, a dynamic microtubule network was simulated for conventional SOFI and JT-SOFI imaging. The overall labeling densities in conventional SOFI and JT-SOFI were set identical to 24 μm^{-1}. 300 frames were collected for conventional SOFI imaging. For JT-SOFI imaging, 100 frames were collected for each channel, which corresponds to 300 frames for three channels. The dynamic motion was simulated through rotating the microtubule network by 0.01 degree during each frame, resulting in 1 degree after JT-SOFI collection and 3 degrees after conventional SOFI collection, the rotation orientation is indicated by the white clockwise arrow in Fig. S13a. Fig. S13a shows the averaged result by averaging 300 frames collected from three channels by joint tagging. Fig. S13b presents the result from conventional SOFI processing by collecting 300 frames using one single channel. Apparently, owing to the dynamic motion of the microtubule network during acquisition, conventional SOFI gives a severely blurred image, as indicated in magnified images in Figs. S13e and S13h. Nonetheless, JT-SOFI image in Fig. S13c presents a much better result. As can be seen, in magnified images indicated in Figs. S13f and S13i, the blurring is almost negligible compared to Figs. S13e and

S13h. Furthermore, the resolution enhancement obtained from JT-SOFI is more significant than that from conventional SOFI. From the cross-sections shown in Fig. S13j, JT-SOFI is able to discriminate the sub-diffraction interleaving structure indicated by the white dashed lines in Figs. S13d, S13e and S13f. However, due to the diffraction limit and limited resolution, the averaged image cannot distinguish this structure. Moreover, conventional SOFI also fails to resolve this structure. This can be ascribed to the severe blurring and reduced resolution improvement caused by the insufficient imaging speed and incompetent high labeling density implementation when conventional SOFI is applied to living cell imaging.

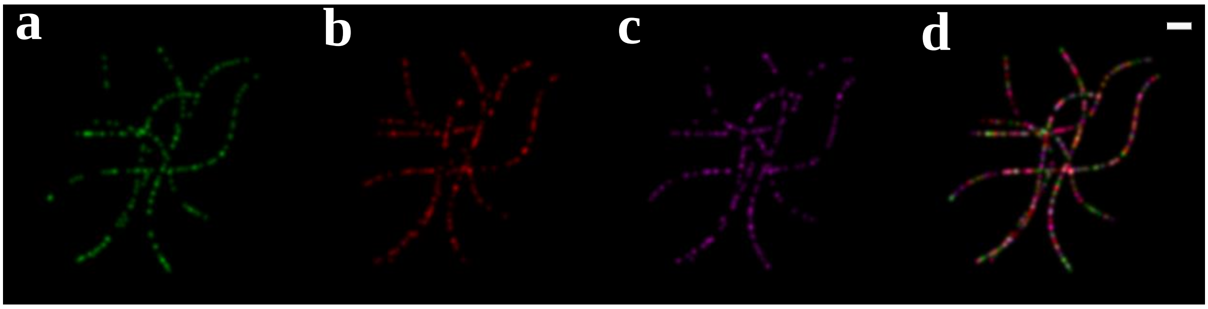

Figure S12 Single frames of the raw images in living cell simulation. (a) The image from the green channel. (b) The image from the red channel. (c) The image from the magenta channel. (d) Merged image of the three channels. ***Scalebar: 1 μm*****.**

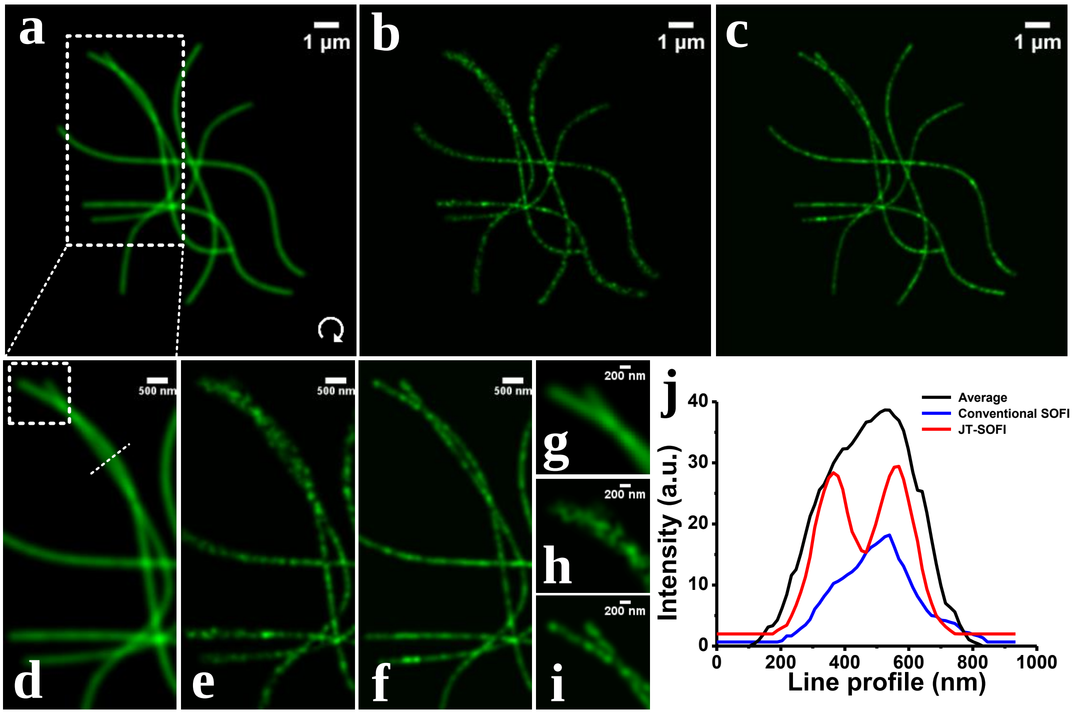

Figure S13 Simulation of real-time imaging of living cells. (a) Average image. (b) Conventional SOFI image. (c) JT-SOFI image. (d-f) Magnified regions from the white boxed regions in (a-c). (g-i) Small regions outlined by the white boxes in (d-f). (j) Cross-sections indicated by the white dashed lines in (d-f).

JT-SOFI imaging reveals the dynamics of lipid rafts tagged with dual-color QDs in living cell.

To demonstrate the potential application of JT-SOFI to living cell imaging, we observed lipid rafts tagged with dual-color QDs in living COS7 cells, and single-particle tracking SOFI (sptSOFI) has been implemented[2]. In this experiment, QD525 and QD655 were jointly tagged to the lipid rafts. In order to simultaneously collect the fluorescence signals from QD525 and QD655 with one single CCD camera, we employed an image splitter to divide the CCD into two independent regions for simultaneous detection of the two channels. The dual-color QDs were simultaneously excited by 488 nm laser. The exposure time of the camera was set at 18 ms. The time-lapse sequences were obtained by continuously capturing the two channel images for hundreds of frames. And each 100 frames were extracted for reconstructing a SOFI image at a particular time point. Figs. S15a-b are the wide-field image and the corresponding 3rd SOFI image from 100 frames in the QD525 channel. Figs. S15c-d are the wide-field image and the corresponding SOFI image from 100 frames in the QD655 channel. As can be seen, the out-of-focus blur can be well rejected and the spatial resolution was enhanced after SOFI processing. Further, we observed the dynamic motion of single QD-tagged lipid raft indicated by the dotted white circles in Figs. S15a-b. Fig. S15e presents five average images of the labeled lipid raft during a time course from 0 to 7.2 seconds. From a merged image of the 1st and 5th images in Fig. S15e, it is difficult to elaborate the dynamic movement of the lipid raft. However, from the SOFI counterparts shown in Fig. S15f, the dynamic movement of the labeled lipid raft can be evidently observed. We estimated that the lipid raft underwent a movement of ~100 nm along horizontal axis during 7.2 s, which was unavailable from the average image sequences. Furthermore, with the implementation of JT-SOFI, the labeling density can be increased by m-folds, consequently the lipid rafts that can be tracked are increased as well. Figs. S15g and S15h present the average and SOFI results in the QD655 channel. Due to the low SNR of this channel, 200 images were used for 2nd order SOFI processing.

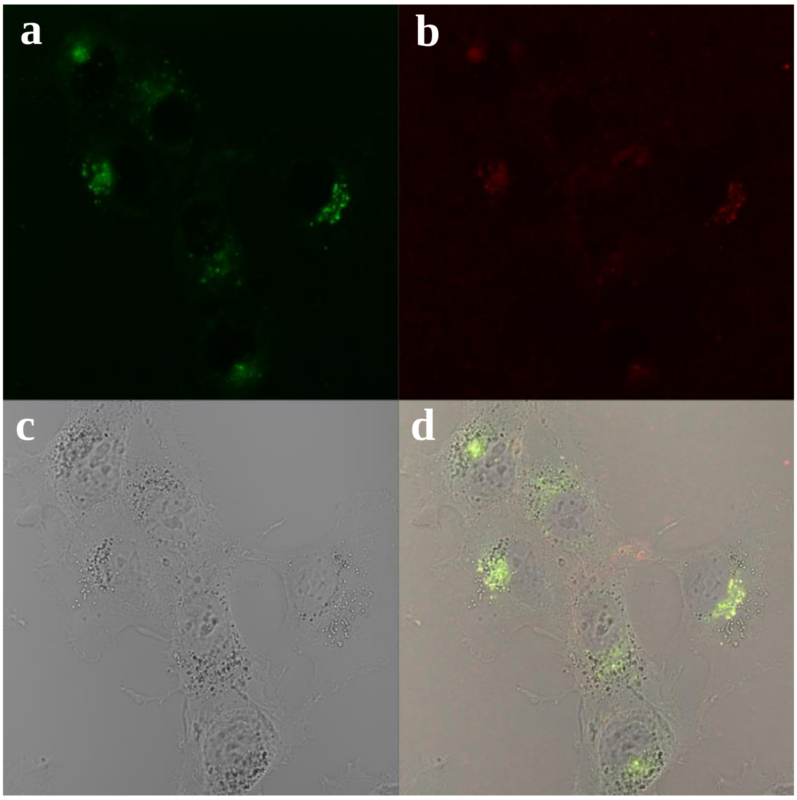

Figure S14 Confocal and bright-field images of the lipid rafts in living COS7 cells. (a) Lipid rafts labeled by QD525. (b) Lipid rafts labeled by QD655. (c) Bright-field image of living COS7 cells. (d) Merged image of (a), (b) and (c).

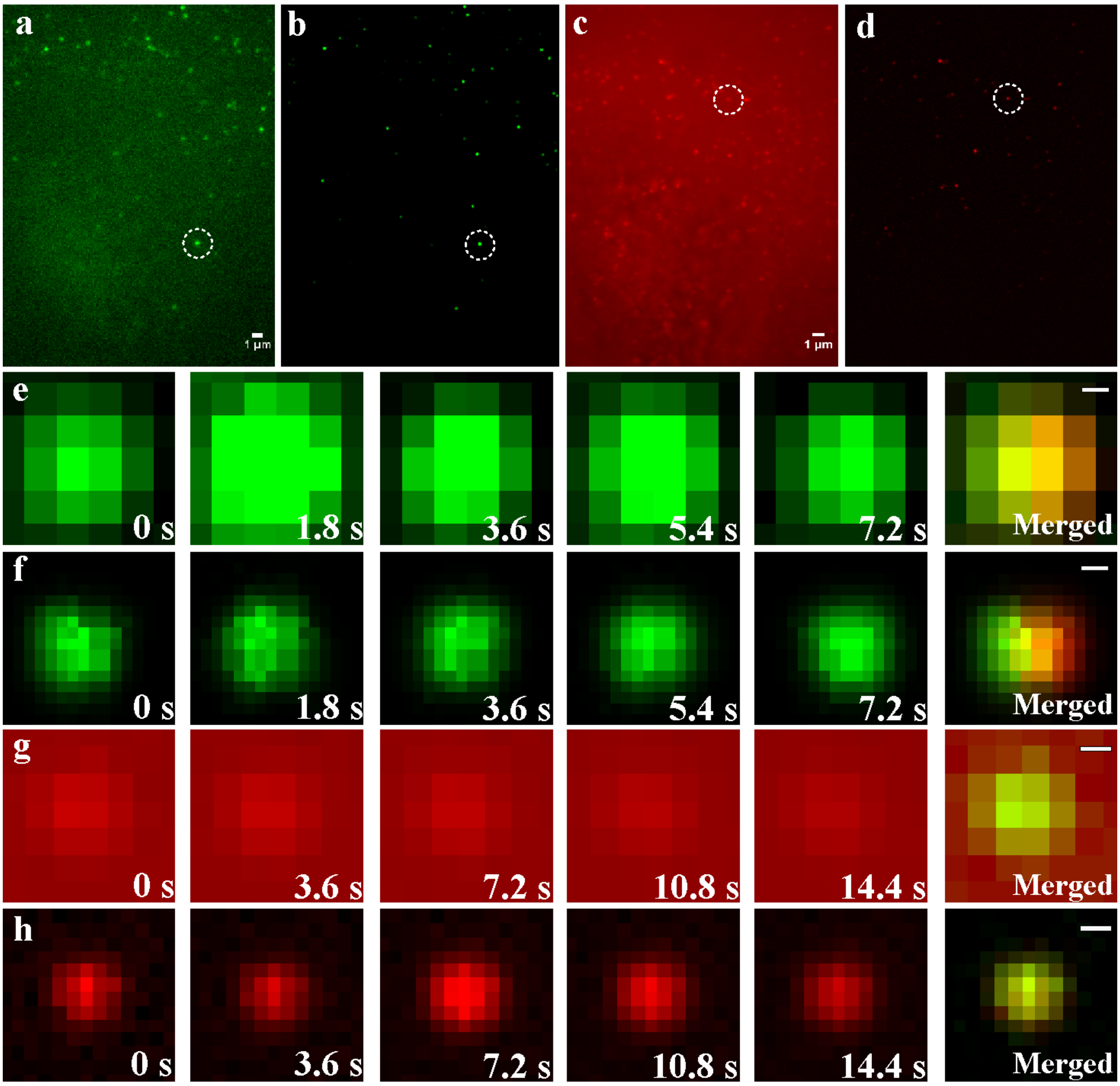

Figure S15 Wide-field and JT-SOFI images of lipid rafts tagged with dual-color QDs in living COS7 cell. (a) Wide-field image of lipid rafts labeled by QD525. (b) 3rd SOFI image of lipid rafts labeled by QD525. (c) Wide-field image of lipid rafts labeled by QD655. (d) 2nd SOFI image of lipid rafts labeled by QD655. *Scale bars in (a,c): 1 µm.* (e,f) Magnified regions indicated by the dotted white circles in (a,b) showing the time-lapse average and SOFI images of a lipid raft. The 6th images in (e,f) are the merged images of the corresponding 1st and 5th images. The color of the 5th images were reassigned as 'red' to distinguish from the 1st images when merged. (g,h) Magnified regions indicated by the dotted white circles in (c,d) showing the time-lapse average and SOFI images of a lipid raft. The 6th images in (g,h) are the merged images of the corresponding 1st and 5th images. The color of the 1st images were reassigned as 'green' to distinguish from the 5th images when merged. *Scale bars in (e-h): 100 nm.*

Image acquisition.

The image acquisition of the lipid rafts in live COS7 cells (African green monkey fibroblast) was carried out on a wide-field microscope (Olympus IX71) with an oil objective (100x, Numerical aperture: 1.49). A laser with the wavelength of 488 nm was used for simultaneously exciting the fluorescence of both QD525 and QD655. The images were captured by an EMCCD (Andor iXon Ultra), which was split into two regions by an image splitter (OptoSplit) for simultaneous detection of dual-color fluorescence signals. The exposure time of the EMCCD was set at 18 ms.

Live cell culture and labeling protocol of the lipid rafts by quantum dots.

COS7 cells were seeded on a glass-cover-slip-bottomed cell culture dish (NEST) for overnight growth at 37 ℃ under 5% CO2. For labeling the lipid rafts, COS7 cells were first incubated with 1 μg/ml cholera toxin subunit B (recombinant), biotin-XX conjugate (Invitrogen) for 30 min before a 10 min 0.5 nM Qdot® 525 and 655 streptavidin conjugate (Invitrogen) incubation, then washed the cells by PBS three times and added the fresh culture medium. After culturing for 0.5-1 h, the endocytosis of the lipid rafts labeled by quantum dots was detected.

TABLE S1 A list of the mean microtubule track lengths in different channels.

	Mean track length (μm)	Maximum track length (μm)
QD525	0.736	22.528
QD625	1.128	48.750
QD705	0.842	28.544
Multi-color	2.283	106.698

TABLE S2 Comparison of the conventional diffraction limited and super-resolution microscopy techniques.

	Wide-field	Confocal	JT-SOFI	STED	SIM	PALM /STORM
Imaging speed	Video-rate	Video-rate	seconds per frame	seconds per frame	seconds per frame	minutes per frame
Resolution (nm)	~ 250	~ 200	~ 80	< 50	~ 120	~ 20
Cost	Low	Moderate	Low	High	Moderate	Moderate

In Fig. 2p, we simulated the visibility of SOFI reconstructed images versus labeling densities. Here, the visibility is defined as

$$v = \frac{I_{max,1} - I_{min}}{2\left(I_{max,1} + I_{min}\right)} + \frac{I_{min} - I_{max,2}}{2\left(I_{min} + I_{max,2}\right)}. \quad (1)$$

where $I_{max,1}$, $I_{max,2}$ and I_{min} are the local maximum and minimum of the corresponding ROI.

The custom-written Matlab program code for analyzing the numbers and lengths of the continuous microtubule skeletons.

```
%% Calculate the numbers and lengths of the input binary images
function [y,leng] = calNum( img)
% img is a binary image matrix;
% y is the number of lines except background
[L, num] = bwlabel( img, 8);
L1 = L.* img;
temp = num;
for kk = 1:num
    index = find( L1 == kk);
    if( length(index) == 0)
        temp = temp - 1;
    end
end
y = temp;
for kk = 1:num
    index = find( L1 == kk);
    leng(kk)=length(index);
end

%% Plot histograms for the skeletonized images
close all
clear all
clc
A1 = imread('525_skeleton.tif');
B1 = imread('multi_color.tif');
A2 = im2bw(double(A1));
B2 = im2bw(double(B1));
[y1,leng1] = calNum( A2);
[y2,leng2] = calNum( B2);
figure
x1 = 1:1:4000;
```

```
[N1,X1]=hist(leng1,x1);
axis([-10 4000 0 75])
h = findobj(gca,'Type','patch');
set(h,'FaceColor','r','EdgeColor','w')
figure
x2 = 1:1:4000;
[N2,X2]=hist(leng2,x2);
axis([-100 4000 0 70])
h2 = findobj(gca,'Type','patch');
set(h2,'FaceColor','r','EdgeColor','w')
```

SUPPORTING REFERENCES

1. Guizar-Sicairos M, Thurman ST, Fienup JR. Efficient subpixel image registration algorithms. *Opt. lett.* 33, 156-158 (2008).

2. Manley S*, et al.* High-density mapping of single-molecule trajectories with photoactivated localization microscopy. *Nat. Methods* 5, 155-157 (2008).
